# Lac water extract inhibits IFN-γ signaling through JAK2-STAT1-IRF1 axis in human melanoma

**DOI:** 10.1039/c8ra02955e

**Published:** 2018-06-13

**Authors:** Luhui Li, Satoru Yokoyama, Na Han, Yoshihiro Hayakawa

**Affiliations:** Division of Pathogenic Biochemistry, Institute of Natural Medicine, University of Toyama 2630 Sugitani Toyama 930-0194 Japan yokoyama@inm.u-toyama.ac.jp; Development and Utilization Key Laboratory of Northeast Plant Materials, School of Traditional Chinese Materia Medica, Shenyang Pharmaceutical University Shenyang 110016 China

## Abstract

Interferon-γ (IFN-γ) is a cytokine that plays an important role in the host defense of infectious diseases and in immune surveillance during tumor development; however, it has adverse effects in the pathogenesis of autoimmune diseases and in immunosuppressive microenvironments, promoting the immunoevasion of cancer cells. In this study, we identified lac water extract (Lac) as a candidate that can suppress IFN-γ signaling amongst 112 types of natural products, using PD-L1 promoter as a readout for the IFN-γ signaling. Moreover, we determined that Lac inhibits IFN-γ-induced PD-L1 and MHC class I expression on the cell surface in melanoma cells, both of which have been identified as the downstream molecules of IFN-γ signaling. We also determined that Lac inhibited the JAK2-STAT1-IRF1 pathway. Finally, we identified laccaic acids, encompassing laccaic acid A, B, C, and E, as the active components in Lac that inhibit IFN-γ signaling. Collectively, the laccaic acids are lead compounds for a novel inhibitor that targets the JAK2-STAT1-IRF1 pathway for diseases caused by the aberrant activation of IFN-γ signaling.

## Abstract 2

Interferon-γ (IFN-γ) is a cytokine that plays an important role in the host defense of infectious diseases and in immune surveillance during tumor development.

## Graphical abstract

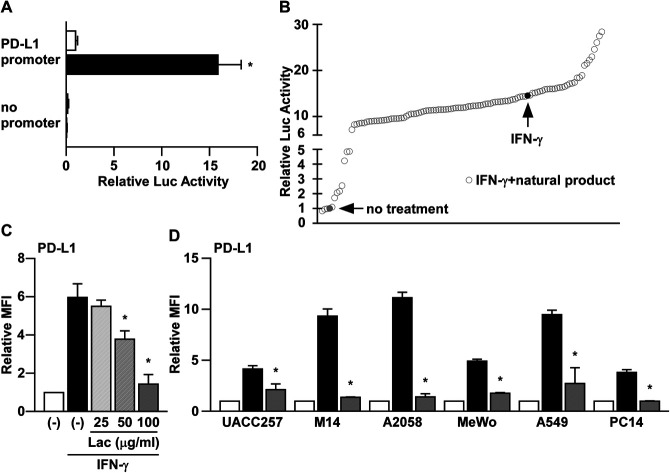

## 1 Introduction

Interferon-γ (IFN-γ) is a pro-inflammatory cytokine, which is secreted by a population of immune cells, such as Th1 cells, cytotoxic T cells, macrophages, and natural killer cells. IFN-γ plays a beneficial role in the host defense of infectious diseases; however, it has adverse effects in the pathogenesis of autoimmune diseases, such as multiple sclerosis,^1^ systemic lupus erythematosus,^2^ type I diabetes,^3^*etc.* Although IFN-γ has been regarded as an important cytokine in immune surveillance during tumor development, accumulating evidence suggests that IFN-γ can also drive an immunosuppressive microenvironment to promote the immunoevasion of pathogens or cancer cells. For instance, it has been reported that cancer cells persistently exposed to IFN-γ acquire resistance to natural killer cells, therefore allowing cancer cells to proliferate or metastasize.^4,5^ IFN-γ has also induced PD-L1 expression by JAK/STAT signaling in various types of tumor and led the escape from immune surveillance.^6–11^ In addition, a complication of the therapeutic use of IFN-γ in melanoma patients has been indicated in clinical trials, where patients with IFN-γ were observed to have worse clinical outcomes than those without IFN-γ.^12–14^ Collectively, the control of IFN-γ and its signaling pathway should be an effective target for developing a new therapy for inflammatory diseases.

In this study, we identified lac water extract (Lac) as a candidate to suppress IFN-γ signaling amongst 112 types of natural products, using PD-L1 promoter as a readout for the IFN-γ signaling. Alongside this, we determined that Lac inhibits IFN-γ-induced PD-L1 and MHC class I expression on the cell surface in melanoma cells, both of which have been identified as the downstream molecules of IFN-γ signaling. We also identified that Lac inhibits IFN-γ signaling through the JAK2-STAT1-IRF-1 axis and that its mechanism is different from that of the JAK inhibitor, baricitinib. Finally, laccaic acids have been identified as the major components of Lac and also as active components to suppress IFN-γ signaling.

## 2 Materials and methods

### 2.1 Reagents

Recombinant human IFN-γ was purchased from Pepro Tech (NJ, USA). Gefitinib, the epidermal growth factor receptor-associated tyrosine kinase inhibitor, was purchased from Cayman Chemical (MI, USA). The lac water extract (Lac) was preserved in the institute of Natural Medicine, University of Toyama. The laccaic acids were purchased from TCI (Tokyo, Japan). Baricitinib was purchased from MedChem Express (Tokyo, Japan).

### 2.2 Cell cultures

PC-9 was a gift from Dr Katsuyuki Kiura (Okayama University, Okayama, Japan). Human melanoma cell lines, UACC257, A2058, M14, and MeWo, and human lung cancer cells, A549, were obtained from American Type Culture Collection (ATCC). All cells were cultured in RPMI-1640 medium (Nissui Seiyaku, Tokyo, Japan) with 10% fetal bovine serum (FBS, Nichirei Biosciences, Tokyo, Japan), 1 mM l-glutamine (Life Technologies, Gaithersburg, MD, USA), 100 U ml^−1^ penicillin, and 100 μg ml^−1^ streptomycin in a humidified atmosphere of 95% air and 5% CO_2_ at 37 °C.

### 2.3 Dual-luciferase assay

The human PD-L1 promoter region was amplified from human genomic DNA and subcloned into the pGL3 basic vector (Promega, Madison, WI, USA). UACC257 cells were co-transfected with each reporter plasmid and pRL-CMV *Renilla* control vector for 4–6 h using Lipofectamine 2000 (Life Technologies) according to the manufacturer's protocol, and replaced with fresh medium. The cells were further cultured with human recombinant IFN-γ (250 ng ml^−1^) just after the treatment of crude drugs/herbs (final 100 μg ml^−1^) for 24 h and lysed with passive lysis buffer. The lysates were assayed by dual-luciferase reporter assay system (Promega). The reported results are the averages of two independent experiments, normalized for transfection efficiency using *Renilla* luciferase activity.

### 2.4 Flow cytometry

The cells were treated with Lac, baricitinib, gefitinib, laccaic acids, or IFN-γ at the indicated concentrations and stained with PE-conjugated anti-human PD-L1 cloneM1H1 (Bioscience, CA, USA) or FITC-conjugated anti-human HLA-ABC (BD Biosciences, MI, USA). The cells were assessed using a FACSCanto II (BD Biosciences). The data was analyzed using the FlowJo software (TreeStar, OR, USA), and the PD-L1 levels or HLA-ABC levels were determined by calculating the median fluorescence intensity (MFI).

### 2.5 Western blot analysis

Whole cell lysates were prepared as previously described.^15^ The primary antibodies used were pJAK2 (Tyr1007/1008), JAK2, pSTAT1 (Tyr701), STAT1 (Cell Signaling Technology, Beverly, MA, USA), IRF-1 and β-actin (Santa Cruz Biotechnology, Santa Cruz, CA, USA).

### 2.6 HPLC-ESI-MS/MS analysis

Lac water extracts were dissolved in 50% methanol to a concentration of 1 mg ml^−1^. Laccaic acid was dissolved in 50% acetonitrile to a concentration of 1 mg ml^−1^. After being filtered with a 0.45 μm hydrophilic PTFE membrane, a 10 μl sample was subjected to LC-MS analysis. LC-MS analysis was performed with a Thermo Scientific™ Accela HPLC system coupled to an LTQ Orbitrap XL hybrid Fourier-transform mass spectrometer (Thermo Fisher Co., San Jose, CA, United States). A Zorbax Extend-C18 HPLC column (4.6 mm × 250 mm, 5 μm, Agilent, USA) was used for HPLC analysis at a flow rate of 500 μl min^−1^ at 40 °C. A gradient elution system composed of 0.1% aqueous formic acid (v/v) (A) and methanol (B) was used as follows: 0–2 min, 15% B; 2–8 min, 15–45% B; 8–25 min, 45–75% B, 25–27 min, 75–80% B, 27–29 min, 80–95% B, 29–30 min, 15% B, 30–35 min, 15% B. The MS conditions were set as follows: negative ESI mode, spray voltage 4.5 kV, capillary voltage 40.0 kV, tube lens 150 V, capillary temperature 270 °C, sheath gas flow rate 50 units, aux gas flow rate 10 units, and scan range *m*/*z* 50–2000. A polytyrosine solution was used for instrument calibration before each experiment.

### 2.7 Statistical analysis

The statistical significance was calculated using the Excel software (Microsoft). More than three means were obtained using the one-way or two-way analysis of variance (ANOVA) with Bonferroni correction, and two means were obtained using unpaired Student's *t*-test. *p* < 0.05 was considered to be statistically significant.

## 3 Results

### 3.1 Lac water extract suppresses IFN-γ-induced PD-L1 expression in melanoma

**Fig. 1:**
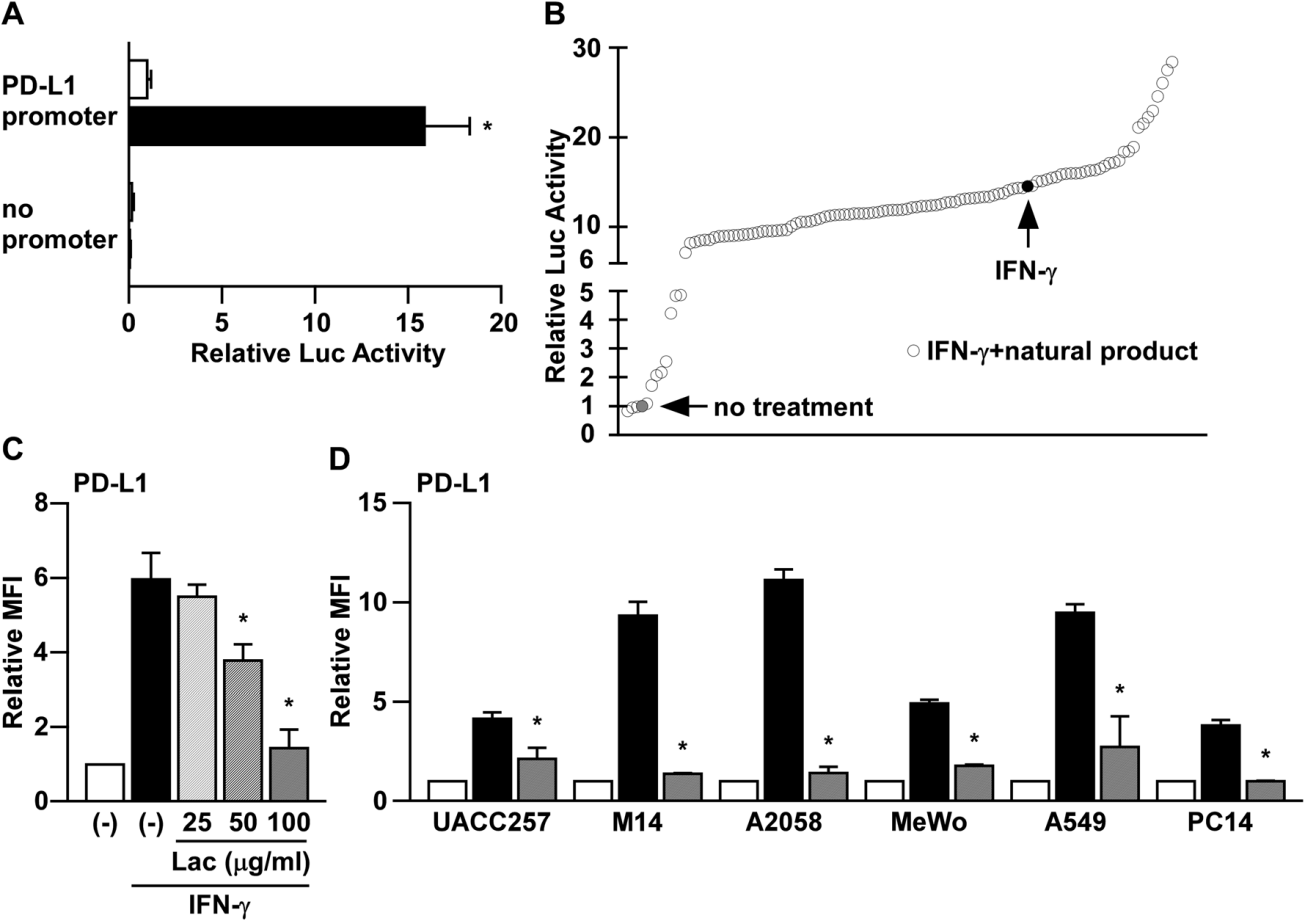
Herbal medicines suppress IFN-γ-induced PD-L1 promoter activity. (A) UACC257 cells were co-transfected with the reporter plasmid containing human *PD-L1* promoter sequences (PD-L1 promoter) or no promoter sequences (no promoter) upstream of the *firefly* luciferase gene with the reference reporter plasmid containing *Renilla* luciferase gene. After 24 hours of IFN-γ (250 ng ml^−1^) treatment, the cell lysates were subjected to dual-luciferase assay. The luciferase activity was normalized to that of the vehicle control in the *PD-L1* promoter. Data are the means ± S.D. of three independent experiments. *p < 0.01 by two-way ANOVA followed by the Bonferroni *post-hoc* test compared with vehicle control and IFN-γ. (B) Using the PD-L1 reporter plasmid in UACC257 cells, the water extracts of natural products were screened. After the addition of the water extracts of natural products at 100 μg ml^−1^, the cells were incubated with IFN-γ (250 ng ml^−1^) for 24 hours. The luciferase activity of each natural product was normalized to that of the vehicle control. Data are the mean of two independent experiments. (C) UACC257 were treated with Lac at the indicated dose and human IFN-γ (10 ng ml^−1^) for 24 hours. The PD-L1 expression was determined as the median fluorescence intensity (MFI) by flow cytometry analysis. Relative PD-L1 expression was normalized to the MFI in non-treated cells. Data are the means ± S.D. of three independent experiments. *p < 0.01 by one-way ANOVA followed by the Bonferroni *post-hoc* test compared with IFN-γ-treated cells. (D) Melanoma cells (UACC257, M14, A2058, and MeWo) and lung cancer cells (A549 and PC-14) were treated with Lac (10 μg ml^−1^) and human IFN-γ (10 ng ml^−1^) for 24 hours. Other conditions are similar to those in Fig. 1C.

It has been reported that PD-L1 expression is induced by IFN-γ in human melanoma cells at the transcriptional level.^8^ To screen the natural products that inhibit the IFN-γ signaling pathway, we firstly established a reporter assay system using *PD-L1* promoter in human melanoma cells. Consistent with the previous reports,^8^ the *PD-L1* promoter activity was significantly increased by IFN-γ treatment in UACC257 cells (Fig. 1A). Next, using this reporter assay system, we tried to identify water extracts of natural products that inhibit IFN-γ-induced PD-L1 promoter activity. Amongst 112 water extracts of natural products, we identified that lac extract (hereinafter referred to as Lac) shows a potent inhibitory effect on IFN-γ-induced PD-L1 promoter activity (Fig. 1B).

To determine the effects of Lac on IFN-γ-induced PD-L1 expression on the cell surface, we performed flow cytometry analysis. Along with its inhibition of *PD-L1* promoter activity, Lac was found to inhibit IFN-γ-induced PD-L1 expression on UACC257 cells in a dose-dependent manner (Fig. 1C). Lac was also found to inhibit IFN-γ-induced PD-L1 expression in other human melanoma cells (M14, A2058, and MeWo) and lung cancer cells (A549 and PC-14) (Fig. 1D), suggesting that Lac shows its inhibitory effect on IFN-γ-induced PD-L1 expression regardless of cancer cell type. Considering the responsiveness to IFN-γ and Lac, we thereafter used M14 and A2058 cells in the subsequent experiments in order to identify its mechanism of action.

### 3.2 Lac inhibits IFN-γ signaling in melanoma cells

**Fig. 2:**
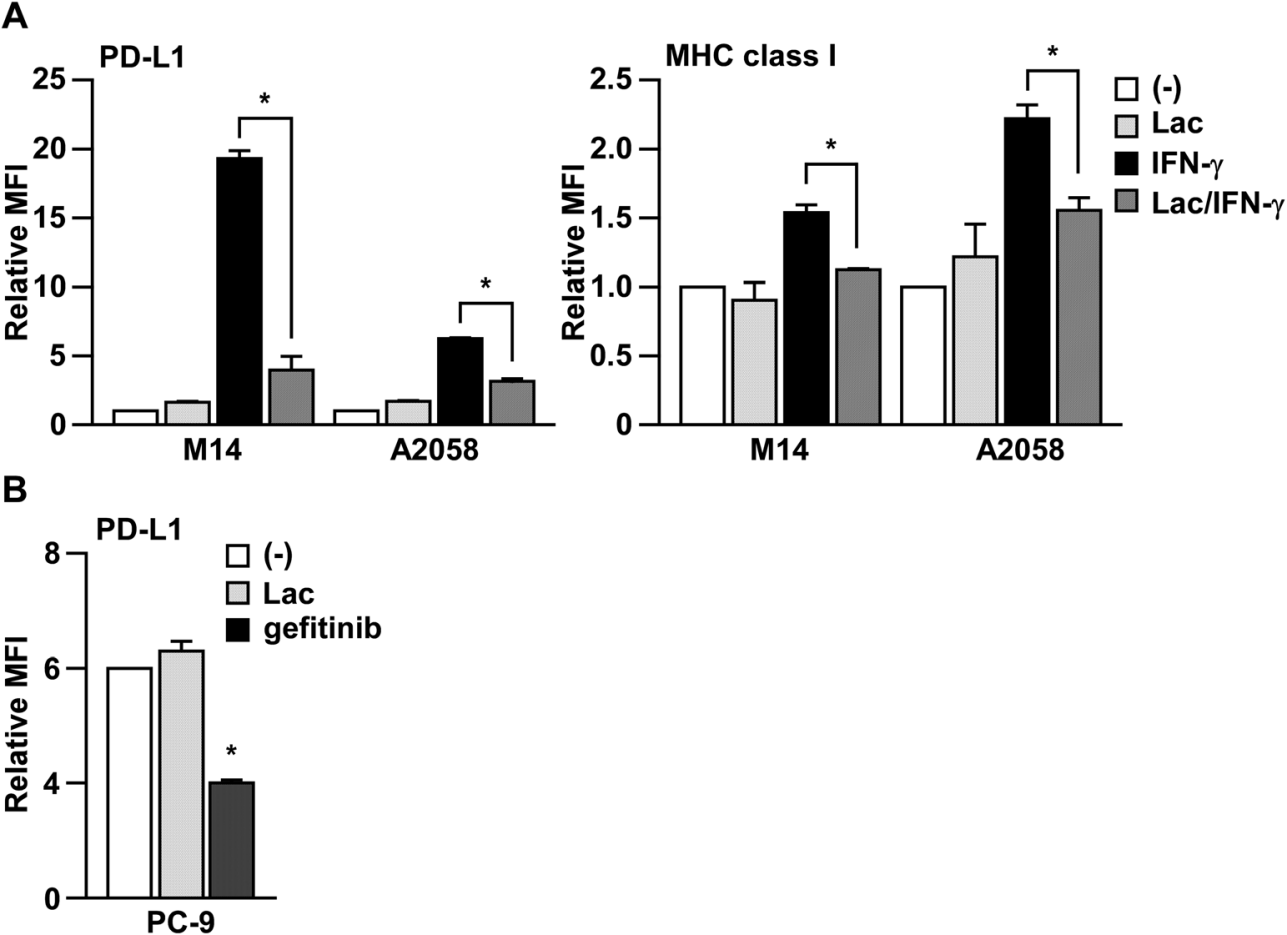
Lac inhibits IFN-γ signaling in melanoma cells. (A) M14 cells were treated with Lac (100 μg ml^−1^) and human IFN-γ (10 ng ml^−1^) for 24 hours. The PD-L1 or MHC class I expression was determined by flow cytometry analysis. Other conditions are similar to those in Fig. 1D. (B) PC-9 cells were treated with Lac or gefitinib, an EGFR inhibitor, for 24 hours. *p < 0.01 by one-way ANOVA followed by the Bonferroni *post-hoc* test compared with non-treated cells.

In order to determine whether Lac inhibits IFN-γ signaling, we next examined the effects of Lac on IFN-γ-induced MHC class I expression on human melanoma cells, which is another typical target molecule induced by IFN-γ.^16^ As shown in Fig. 2A, Lac significantly inhibited the upregulation of MHC class I on M14 and A2058 cells induced by IFN-γ, but did not affect the basal expression of PD-L1 or MHC class I. To further determine the specificity of Lac on IFN-γ signaling to inhibit PD-L1 expression, we examined the effect of Lac on the endogenous PD-L1 expression of PC-9 lung cancer cells, which is mainly regulated by EGFR signaling.^17^ While Lac did not affect PD-L1 expression on PC-9 cells, the EGFR tyrosine kinase inhibitor gefitinib was found to significantly inhibit the endogenous expression of PD-L1 on PC-9 cells (Fig. 2B). These data strongly suggest that Lac selectively inhibits IFN-γ signaling and the expression of its target molecules, PD-L1 and MHC class I, in cancer cells.

### 3.3 Lac inhibits IFN-γ signaling in melanoma cells through the JAK2-STAT1-IRF1 axis

**Fig. 3:**
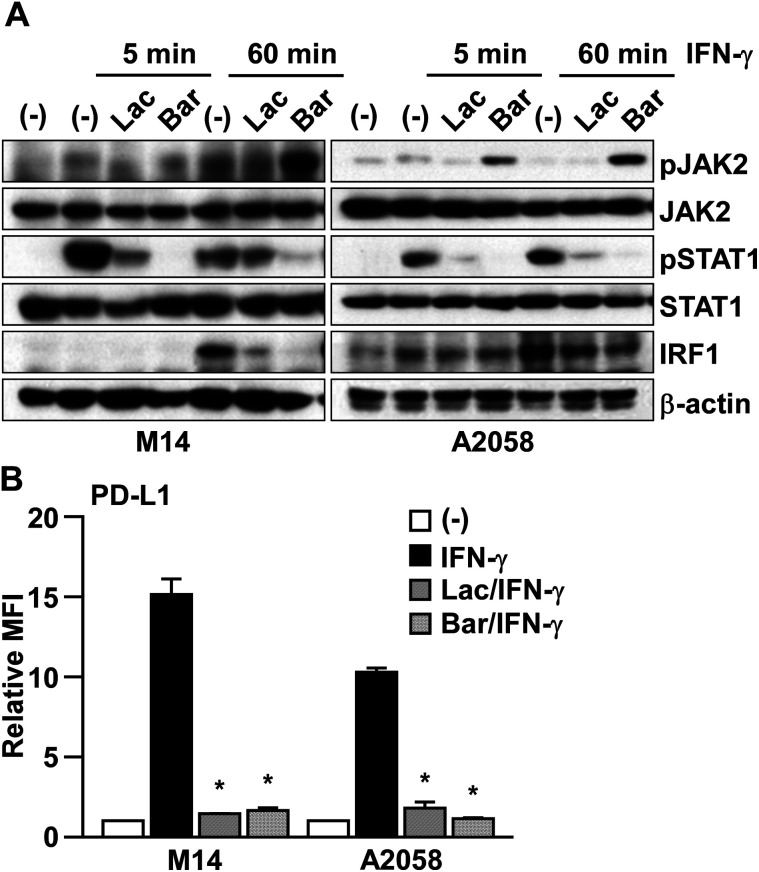
Lac inhibits IFN-γ signaling in melanoma cells through the JAK2-STAT1-IRF1 axis. (A) M14 and A2058 cells were treated with IFN-γ (10 ng ml^−1^) after Lac (100 μg ml^−1^) or baricitinib (0.5 μM) for the indicated times. Whole cell lysates were subjected to western blotting. (B) M14 and A2058 cells were treated with IFN-γ (10 ng ml^−1^) after Lac (100 μg ml^−1^) or baricitinib (0.5 μM) for 24 hours. The PD-L1 expression was determined as the median fluorescence intensity (MFI) by flow cytometry analysis. Other conditions are similar to those in Fig. 1D.

To clarify the mechanism of Lac in inhibiting IFN-γ signaling, we checked the effect of Lac on the downstream molecules, JAK2, STAT1, and IRF1 (Fig. 3A). IFN-γ was found to induce the transient phosphorylation of both JAK2 and STAT1 at 5 min post treatment, and subsequently induced the expression of IRF1 at 60 min post treatment in M14 and A2058 cells. Lac inhibited IFN-γ-induced JAK2 and STAT1 phosphorylation and IRF1 expression, while baricitinib, which is an FDA-approved JAK1/2 inhibitor, also inhibited STAT1 phosphorylation and IRF1 expression in M14 and A2058 cells, and JAK2 phosphorylation was induced by baricitinib treatment (Fig. 3A). Considering that both Lac and baricitinib markedly attenuated IFN-γ-induced PD-L1 expression at the protein level on the cell surface (Fig. 3B), Lac inhibits IFN-γ signaling mainly through the JAK2-STAT1-IRF1 axis.

### 3.4 Laccaic acids might be the active components of Lac

**Fig. 4:**
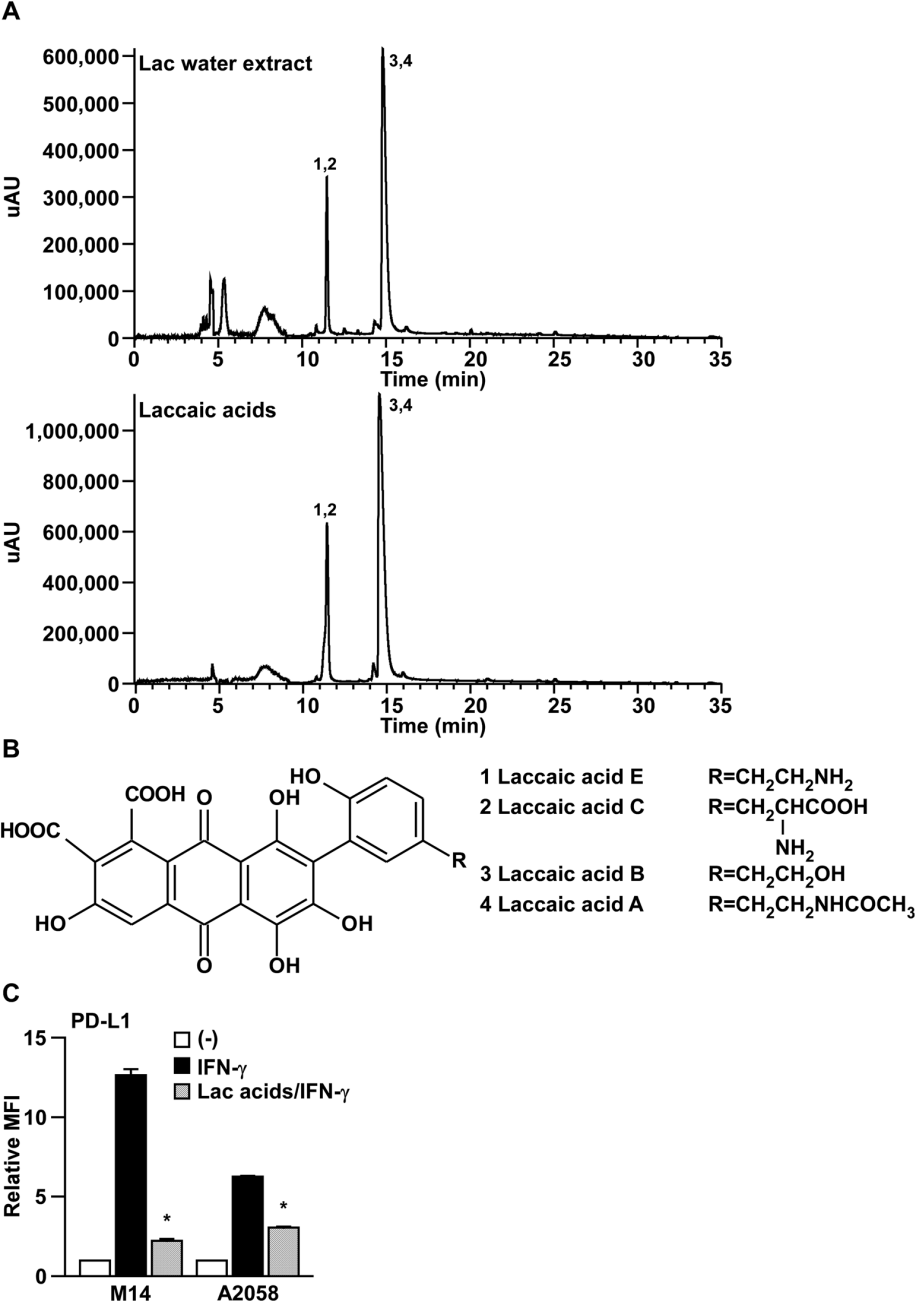
Laccaic acids might be the active components in Lac. (A) HPLC chromatogram of lac water extract or laccaic acids. Their identification was determined from HPLC-ESI-MS/MS analysis. Peaks were identified as follows: (1) laccaic acid E; (2) laccaic acid C; (3) laccaic acid B; (4) laccaic acid A. (B) The structures of laccaic acid A, B, C, and E. (C) M14 and A2058 cells were treated with the laccaic acids (5 μg ml^−1^) and human IFN-γ (10 ng ml^−1^) for 24 hours. PD-L1 expression on the cell surface was determined by flow cytometry analysis. Other conditions are similar to those in Fig. 1D.

To gain insight into the major active components of Lac that inhibit IFN-γ signaling, we performed HPLC-ESI-MS/MS analysis on Lac and laccaic acids. As shown in Fig. 4A and B, and Tables 1 and 2, the major components of Lac were identified as the laccaic acids A, B, C and E, by interpretation of the MS and MS^2^ with relevant references.^18–21^ Importantly, the crude fractions of the laccaic acids significantly suppressed the IFN-γ-induced PD-L1 expression on both M14 and A2058 melanoma cells (Fig. 4C). Considering that similar inhibitory effects were observed by the crude fraction of laccaic acids at lower concentration (5 μg ml^−1^) compared with the Lac water extract at 100 μg ml^−1^, laccaic acids might be the active components of Lac that inhibit IFN-γ signaling.

**Table 1:** LC-MS/MS characterization of Lac water extract (Lac)

No.	RT (min)	Identified compound	[M − H]^−^	MS^2^ ions				Molecular formula
				[M − H–CO_2_]^−^	[M − H–CO_2_–H_2_O]^−^	[M − H–2CO_2_]^−^	[M − H–2CO_2_–H_2_O]^−^	
1	11.48	Laccaic acid E	494.0726	450.0827	432.0725	406.0929	388.2679	C_24_H_17_NO_11_
2	11.48	Laccaic acid C	538.0621	494.1392	476.1940	450.1633	432.0720	C_25_H_17_NO_13_
3	14.81	Laccaic acid B	495.0572	451.1936	433.2540	407.1119	389.1104	C_24_H_16_O_12_
4	14.81	Laccaic acid A	536.0838	492.2119	474.1458	448.1614	430.4215	C_26_H_19_NO_12_

**Table 2:** LC-MS/MS characterization of laccaic acids

No.	RT (min)	Identified compound	[M − H]^−^	MS^2^ ions				Molecular formula
				[M − H–CO_2_]^−^	[M − H–CO_2_–H_2_O]^−^	[M − H–2CO_2_]^−^	[M − H–2CO_2_–H_2_O]^−^	
1	11.44	Laccaic acid E	494.0721	450.0824	432.1078	406.1851	388.2111	C_24_H_17_NO_11_
2	11.44	Laccaic acid C	538.0618	494.0135	476.0724	450.1301	432.0715	C_25_H_17_NO_13_
3	14.57	Laccaic acid B	495.0569	451.2950	433.2215	407.1001	389.1102	C_24_H_16_O_12_
4	14.57	Laccaic acid A	536.0829	492.2720	474.2318	448.2780	430.2499	C_26_H_19_NO_12_

## 4 Discussion

Lac is the secretion of a number of species of lac insects, of which the most commonly cultivated species is *Kerria lacca*. The water extract of lac, known as a lac dye, is a natural pigment, which is commonly used as food colorant.^22^ The main components of lac water extract are laccaic acids, a series of hydroxyanthraquinoid derivatives, namely laccaic acid A, B, C, and E.^21,23^ Although various pharmacological activities of either lac dye or the laccaic acids have been reported, including anti-cancer, anti-microbial and anti-genotoxic activities,^24–26^ the detailed mechanism of the actions of lac dye or the laccaic acids is not well-understood. In this study, we found that lac water extract (Lac) inhibits IFN-γ signaling through the JAK2-STAT1-IRF1 axis. We also identified laccaic acids (A, B, C, E) as the active components of Lac that suppress IFN-γ signaling. Among these laccaic acids, laccaic acid A was found to be the main component of lac water extracts from LC-MS/MS data (Fig. 4A and data not shown), which was also supported by a previous report.^20^ Laccaic acid A has been reported to be a direct inhibitor of DNA methyltransferase1, which may block specific carcinogenesis pathways through alteration of the methylated gene expression;^27^ therefore, we still needed to further investigate the possibility that the inhibition of DNA methyltransferase1 by laccaic acid A may be functionally required for the inhibition of IFN-γ signaling.

Although IFN-γ is regarded as an important cytokine for anti-cancer immune responses,^28^ it is also known to have cancer-promoting aspects.^29–33^ Such tumor-promoting functions of IFN-γ can be supported by the results of IFN-γ trials, in which the melanoma patients treated with IFN-γ showed worse clinical outcomes.^12–14^ Moreover, IFN-γ and chronic inflammation are also known to be involved in promoting tumor development.^34–36^ In accordance with that evidence, it was also reported that the feeding of Lac color inhibits the development of thyroid carcinoma induced by sulfadimethoxine (SDM) in rats,^37^ and that laccaic acid suppresses the SDM-induced T-cell-mediated inflammatory response.^38^ Collectively, Lac might prevent inflammation-induced tumor development through its inhibitory activity in IFN-γ signaling.

IFN-γ is known to activate the JAK-STAT signaling pathway, specifically JAK2 and STAT1.^7,39,40^ By comparing it with baricitinib, a reversible JAK1/2 tyrosine kinase inhibitor, Lac did not show any aberrant activation of JAK2 that can be seen in the baricitinib treatment as a result of the reverse induction of JAK2 phosphorylation at a later time point post IFN-γ treatment (60 min, Fig. 3). These results imply that the mechanism of action of Lac on inhibiting IFN-γ signaling might be at least distinct from that of JAK2 inhibitors. The dysregulation of the JAK/STAT pathway is involved in many diseases, including autoimmune diseases, asthma, diabetes, and cancers, suggesting that the laccaic acids could be lead compounds for a novel inhibitor that targets the JAK2-STAT1-IRF1 pathway for those diseases.

## 5 Conclusions

In conclusion, the present data demonstrate that Lac or laccaic acids inhibit the IFN-γ signaling in melanoma through the JAK2-STAT1-IRF1 axis. This finding indicates that laccaic acids could be lead compounds for a novel inhibitor that targets IFN-γ signaling.

## Conflicts of interest

There are no conflicts of interest to declare.

## Supplementary Material
